# Presence of the *GFI1-36N* single nucleotide polymorphism enhances the response of *MLL-AF9* leukemic cells to CDK4/6 inhibition

**DOI:** 10.3389/fonc.2022.903691

**Published:** 2022-08-08

**Authors:** Jan Vorwerk, Kaiyan Sun, Daria Frank, Felix Neumann, Jana Hüve, Paulina Marie Budde, Longlong Liu, Xiaoqing Xie, Pradeep Kumar Patnana, Helal Mohammed Mohammed Ahmed, Bertram Opalka, Georg Lenz, Ashok Kumar Jayavelu, Cyrus Khandanpour

**Affiliations:** ^1^ Department of Medicine A, Hematology, Hemostaseology, Oncology, and Pneumology, University Hospital Münster, Münster, Germany; ^2^ Fluorescence Microscopy Facility Münster, Institute of Medical Physics and Biophysics, University of Münster, Münster, Germany; ^3^ Evorion Biotechnologies GmbH, Münster, Germany; ^4^ Department of Hematology and Stem Cell Transplantation, West German Cancer Center (WTZ), University Hospital Essen, Essen, Germany; ^5^ Department of Proteomics and Signal Transduction, Max Planck Institute of Biochemistry, Munich, Germany; ^6^ Molecular Medicine Partnership Unit, European Molecular Biology Laboratory (EMBL), Heidelberg, Germany; ^7^ Department of Pediatric Oncology, Hematology, and Immunology, Heidelberg University Hospital, Heidelberg, Germany; ^8^ Hopp Children’s Cancer Center Heidelberg (KiTZ), Heidelberg, Germany; ^9^ Clinical Cooperation Unit Pediatric Leukemia, German Cancer Research Center (DKFZ), Heidelberg, Germany; ^10^ Department of Hematology and Oncology, University Hospital of Schleswig-Holstein, University of Lübeck, Lübeck, Germany

**Keywords:** acute myeloid leukemia, Gfi1, single nucleotide polymorphism, Cdks, Cdk inhibition, palbociclib

## Abstract

The zinc finger protein Growth Factor Independence 1 (GFI1) acts as a transcriptional repressor regulating differentiation of myeloid and lymphoid cells. A single nucleotide polymorphism of *GFI1*, *GFI1-36N*, has a prevalence of 7% in healthy Caucasians and 15% in acute myeloid leukemia (AML) patients, hence most probably predisposing to AML. One reason for this is that GFI1-36N differs from the wildtype form GFI1-36S regarding its ability to induce epigenetic changes resulting in a derepression of oncogenes. Using proteomics, immunofluorescence, and immunoblotting we have now gained evidence that murine *GFI1-36N* leukemic cells exhibit a higher protein level of the pro-proliferative protein arginine N-methyltransferase 5 (PRMT5) as well as increased levels of the cell cycle propagating cyclin-dependent kinases 4 (CDK4) and 6 (CDK6) leading to a faster proliferation of *GFI1-36N* leukemic cells *in vitro*. As a therapeutic approach, we subsequently treated leukemic *GFI1-36S* and *GFI1-36N* cells with the CDK4/6 inhibitor palbociclib and observed that *GFI1-36N* leukemic cells were more susceptible to this treatment. The findings suggest that presence of the *GFI1-36N* variant increases proliferation of leukemic cells and could possibly be a marker for a specific subset of AML patients sensitive to CDK4/6 inhibitors such as palbociclib.

## Introduction

Acute myeloid leukemia (AML) is a malignant disease of the bone marrow (BM) that is characterized by an accumulation of myeloid progenitor cells due to a differentiation block in early phases of hematopoiesis (1). AML is primarily a disease of elderly patients who cannot tolerate intensive chemotherapy and consequently have a poor prognosis (2, 3). Therefore, the development of targeted, personalized therapies sparing non-malignant cells and surrounding tissue is urgently needed.

Growth Factor Independence 1 (GFI1) is a zinc finger protein composed of three domains: an N-terminal SNAIL/GFI1 repressor domain (4), an intermediate domain that seems to be essential for protein-protein interactions (5), and a C-terminal C_2_H_2_ zinc finger domain (6). By recruiting histone-modifying enzymes to its target genes, including *Meis1*, *Hoxa9*, and *Pbx1*, GFI1 is an important regulator of hematopoiesis (7–9). *GFI1-36N* is a single nucleotide polymorphism (SNP) in which the amino acid serine (S) is replaced by asparagine (N) at position 36 of the coding N-terminal region of *GFI1* (10). Thus, there are at least two variants of *GFI1*: a more common wildtype variant called *GFI1-36S* and a rarer variant called *GFI1-36N*. The fact that 7% of the healthy population and up to 15% of all AML patients feature the *GFI1-36N* form underlines that the SNP is widespread in the population and that it contributes to leukemogenesis in a subset of patients (10–12). This is further supported by recent evidence showing that GFI1 interacts with enzymes involved in DNA repair and cell cycle control (5, 13). Such control mechanisms might not be maintained in presence of the *GFI1-36N* SNP.

To this end, we addressed the question of whether *GFI1-36N* leukemic cells exhibit alterations in their cell cycle and whether these provide potential targets for novel therapies. Indeed, we demonstrated that the protein levels of cell cycle-propagating cyclin-dependent kinases (CDKs) and the pro-proliferative protein arginine N-methyltransferase 5 (PRMT5) were increased in murine *GFI1-36N* leukemic cells and that the levels of cyclin-dependent kinase inhibitor proteins (CDKIs) were decreased. Due to substantial differences in CDK4/6 protein levels of both *GFI1* variants, we consequently treated leukemic *GFI1-36S* and *GFI1-36N* cells with the CDK4/6 inhibitor palbociclib and observed that *GFI1-36N* leukemic cells were more sensitive to palbociclib treatment than *GFI1-36S* leukemic control cells.

## Methods

### Generation of knock-in mice

Two mice strains were generated whereby the coding part of the human *GFI1-36S* or *GFI1-36N* variant was inserted into the murine *Gfi1* gene locus, as described before (14). The mice were kept in IVC cages under SPF conditions in the Central Animal Facility (ZTE) of the Faculty of Medicine at the University of Münster. All animal experiments were approved by the local ethics committee of North Rhine-Westphalia (81-02.04.2019.A440).

### Generation of leukemic mice

Leukemic mice were generated by injecting retrovirally transduced *MCSV-MLL-AF9-IRES-GFP* lineage-negative cells into lethally irradiated (7 + 3 Gy) C57BL/6 mice (Jackson Laboratory, Bar Harbor, ME, United States), as described previously (15, 16). After AML development, the leukemic BM cells were harvested and transplanted into sublethally irradiated (3 Gy) C57BL/6 mice. Irradiation was done using the MultiRad225 irradiation system (Precision X-Ray, Madison, CT, United States). To prevent infections after BM transplantation, enrofloxacin (Bayer, Leverkusen, Germany) was added to the drinking water of the mice. The leukemic BM cells of the secondary transplanted mice were used for further transplantations or for the experiments described below.

### Immunofluorescence

After having been washed twice in PBS, 0.5×10^6^ cells were adhered to ImmunoSelect adhesion slides (Squarix, Marl, Germany) for 15 min. They were then fixed with 3% PFA + 2% sucrose in PBS for 15 min, permeabilized with 100 mM Tris + 50 mM EDTA + 0.5% Triton X-100 in ddH_2_O for 10 min, and blocked with 2% BSA in PBS overnight at 4 °C. The GFI1 (M01, Abnova, Taipei, Taiwan) primary antibody was diluted 1:37 in 2% BSA in PBS, the PRMT5 (ab109451, Abcam, Cambridge, United Kingdom), CDK4 (sc-70831, Santa Cruz Biotechnology, Dallas, TX, United States), and CDK6 (sc-7941, Santa Cruz Biotechnology) primary antibodies were diluted 1:50 in 2% BSA in PBS. The slides with the added antibodies were incubated for 120 min at RT. They were then washed three times in PBS and stained with 1:500 goat anti-mouse Alexa Fluor 555 (ab150114, Abcam), 1:2,000 goat anti-rabbit IgG Cy5 (ab6564, Abcam), or 1:700 donkey anti-rabbit Alexa Fluor 488 (ab150065, Abcam) secondary antibodies for 90 min at RT. The slides were washed once in PBS and incubated in 50 ng/ml DAPI (Sigma-Adrich, St. Louis, MO, United States) in PBS for 10 min. Finally, the cells were rewashed and embedded in Dako Fluorescence Mounting Medium (Agilent Technologies, Santa Clara, CA, United States). Z-stacks of the samples were acquired using a Leica TCS SP8 confocal laser scanning microscope with an HC PL APO 63×/1.40 oil CS2 objective and Leica HyD hybrid detectors running on the LAS X version 3 software (Leica, Wetzlar, Germany). Maximum intensity Z-projections of each channel and counting of foci were done using the software ImageJ (National Institutes of Health, Bethesda, MD, United States).

For better visualization of colocalization in non-leukemic cells, we chose to display GFI1 foci of non-leukemic cells in red, whereas they appear yellow in leukemic cells. In both cells, staining was done with the same Alexa Fluor 555 secondary antibody. Because non-leukemic cells do not express *GFP*, the PRMT5 foci were visualized using the green Alexa Fluor 488 secondary antibody, whereas they were stained with the red Cy5 secondary antibody in the *GFP*-expressing leukemic cells.

### Immunoblotting

For treatment experiments, cells were incubated for 24 h with palbociclib (Selleck Chemicals, Houston, TX, United States) in ddH_2_O. Cells were washed in PBS and incubated for 30 min on ice in a Halt protease inhibitor cocktail (Thermo Fisher Scientific, Waltham, MA, United States) dissolved in PhosphoSafe extraction reagent (Merck Millipore, Burlington, MA, United States). During incubation, the suspension was vortexed every 5 min. The cells were then centrifuged at 13,000 g for 30 min. The protein concentration of the lysate was determined by BCA assay (Thermo Fisher Scientific). 3.75 μl NuPAGE LDS sample buffer (Thermo Fisher Scientific) and 1.5 μl NuPAGE reducing agent (Thermo Fisher Scientific) were added to 20 μg proteins and heated at 95 °C for 5 min. The samples were separated at 100 V for 90 min on a 10% polyacrylamide gel and then electroblotted under the same conditions onto an Immobilion PVDF membrane (Merck Millipore). The membranes were blocked in 5% milk in TBS-T for 120 min. The PRMT5 antibody (ab109451, Abcam) was diluted 1:10,000, the CDK4 antibody (sc-70831, Santa Cruz Biotechnology) 1:1,000, the CDK6 antibody (sc-7941, Santa Cruz Biotechnology) 1:200, the RB1 antibody (D20, Cell Signaling Technology, Danvers, MA, United States) 1:1,000, the phospho-RB1 antibody (D20B12, Cell Signaling Technology) 1:1,000, and the actin antibody (sc-8432, Santa Cruz Biotechnology) 1:2,000 in 5% milk in TBS-T. The membranes with the antibodies were incubated on a tube roller at 4 °C overnight. The following day the membranes were washed three times for 5 min in TBS-T. The goat anti-mouse IgG-HRP (1031-05, SouthernBiotech, Birmingham, AL, United States) and mouse anti-rabbit IgG-HRP (sc-2357, Santa Cruz Biotechnology) secondary antibodies were diluted 1:5,000 in 5% milk in TBS-T. The membranes were incubated in the antibody solutions for 120 min at RT and then washed three times for 15 min in TBS-T. Detection was performed in Amersham Imager 600 (Cytiva, Marlborough, MA, United States) using the ECL substrates Lumi-Light (Roche, Basel, Switzerland) or RadiancePlus (Azure Biosystems, Dublin, CA, United States).

### Proteome analysis

#### Label-free proteome quantification

Cells were freshly collected, washed in PBS, and lysed in 1% SDC buffer (1% SDC, 100 mM Tris, 40 mM CAA, and 10 mM TCEP). Following lysis, samples were incubated for 20 min on ice, heated at 95 °C, and sonicated for 5 min as described before (17). The samples were digested in 1:100 LysC for 120 min, followed by 1:100 trypsin overnight at 37 °C. To stop digestion, propan-2-ol + 1% TFA was added. The peptides were then desalted on SDB-RPS StageTips, washed first with propan-2-ol + 1% TFA and second twice with 0.2% TFA. The purified peptides were rinsed using 60 µl elution buffer (80%, 1.25% NH_4_OH) and resuspended in mass spectrometry (MS) loading buffer (3% ACN, 0.3% TFA). Until measurement, the samples were stored at -20 °C.

#### Single-shot liquid chromatography-MS/MS measurement

An EASY-nLC1000 nanoflow HPLC linked to a Q Exactive HF-X Hybrid Quadrupole-Orbitrap mass spectrometer (Thermo Fischer Scientific) using a nano-electrospray ion source was used for measurement. An in-house prepared column with a diameter of 75 µM was packed with 1.9 µM C_18_ ReproSil particles (Dr. Maisch, Ammerbuch, Germany) and was kept at a constant temperature of 60 °C. Peptide amount equal to 450 ng was separated using a binary buffer system of buffer A (0.1% methanoic acid) and buffer B (60% ACN + 0.1% methanoic acid) at a flow rate of 300 nl/min. The peptides were eluted with a gradient of 30% buffer B for 95 min, which was then increased to 60% buffer B for 5 min. This was followed by a rapid increase to 95% for 5 min and a decrease to 5% for 5 min. The samples were analyzed with one full scan at a target of 3e^6^ ions (300–1,650 *m/z*, *R* = 60,000 at 200 *m/z*) followed by top15 MS/MS scans with high-energy collisional dissociation-based fragmentation (target: 1e^5^ ions, maximum fill time: 28 ms, isolation window: 1.4 *m/z*) and detected in Orbitrap at a resolution of 15,000 at 200 *m/z*.

#### MS data processing

Processing of raw files was done using MaxQuant version 1 (18), supported by the Andromeda search engine. Data were searched for peptides with the help of a target-decoy approach with a reverse UniProt FASTA database with an FDR of < 1%. The minimum peptide length was set to seven amino acids. No more than two missed cleavages were allowed. If two proteins could not be distinguished by unique peptides, they were attributed to the same protein groups. The MaxLFQ algorithm (19) was used for label-free quantification. For identifying peptides based on mass accuracy and retention times the “match between runs” feature was activated. The MaxQuant output table was analyzed in Perseus version 2 (20).

### Cell viability assay

5×10^3^ cells were incubated with different concentrations of palbociclib dissolved in ddH_2_O. After 72 h, the cell suspensions were added to an equal volume of CellTiter-Glo (Promega, Madison, WI, United States) and mixed for 30 min on an orbital shaker. Cell viability was measured in Victor X3 multimode plate reader running on PerkinElmer 2030 version 4 software (PerkinElmer, Waltham, MA, United States) according to the manufacturer’s instructions.

### Colony-forming unit assay

7.5×10^3^ cells and different concentrations of palbociclib in ddH_2_O were added to 1 ml MethoCult M3434 methylcellulose-based medium (Stemcell Technologies, Vancouver, BC, Canada). The suspension was mixed by vortexing and plated into 6-well plates using 16 G blunt-end needles (Stemcell Technologies). After 11 d of incubation, colonies were counted using a Zeiss Axio Vert.A1 inverted microscope (Zeiss, Oberkochen, Germany).

### Apoptosis assay

0.5×10^6^ cells were incubated with palbociclib in ddH_2_O for 24 h at 37°C. The following day, cells were washed once in PBS. The cell pellet was resuspended in 100 μl Annexin V binding buffer (Thermo Fisher Scientific) containing 0.5 μl Annexin V-APC (BioLegend, San Diego, CA, United States) and incubated for 10 min at RT. 100 μl Annexin V binding buffer containing 0.5 μl propidium iodide (Thermo Fisher Scientific) was added and incubated for 10 min at 4°C. Analysis was performed in an Attune NxT acoustic focusing cytometer using Attune NxT version 3 software (Thermo Fisher Scientific).

### Cell cycle assay

Cell cycle assay was performed using a NucleoCounter NC-250 two-step cell cycle analysis kit (ChemoMetec, Allerod, Denmark) according to the manufacturer’s instructions. Briefly, cells were seeded into 12-well plates at the density of 0.5×10^6^/ml in IMDM (Thermo Fisher Scientific) + 20% FCS (PAN-Biotech, Aidenbach, Germany). Cells were incubated with palbociclib in ddH_2_O, harvested after 24 h, and washed once in PBS. Pellets were resuspended in 100 μl lysis buffer containing 10 μg/ml DAPI and incubated for 5 min at 37°C. 100 μl of stabilization buffer was added to measure the samples in NucleoCounter NC-250 (ChemoMetec). Data were analyzed using ModFit LT version 5 software (Verity Software House, Topsham, ME, United States).

### Statistical analysis

The statistical evaluation of the experiments was carried out with GraphPad Prism version 6 software (GraphPad Software, San Diego, CA, United States) by performing two-sided unpaired t-tests between the *GFI1-36N* and *GFI1-36S* leukemic cells. A result was rated significant under the condition *p* ≤ 0.05 (*p* ^*^ ≤ 0.05, *p* ^**^ ≤ 0.01, *p* ^***^ ≤ 0.001, *p* ^****^ ≤ 0.0001).

## Results

### *GFI1-36N* leukemic cells are associated with an increased PRMT5 level

**Figure 1:**
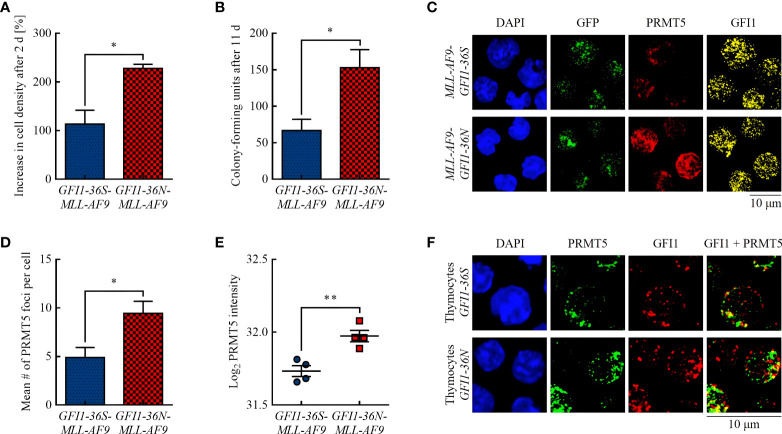
*GFI1-36N* leukemic cells are associated with higher proliferation and an increased protein level of the arginine-methyltransferase PRMT5. **(A)** Cell proliferation after 2 d. **(B)** CFUs after 11 d. **(C)** PRMT5 and GFI1 foci. Blue (DAPI) = nucleus, green (GFP) = leukemic cells, red (Cy5) = PRMT5, yellow (AF555) = GFI1. **(D)** Quantification of PRMT5 foci. **(E)** PRMT5 protein intensity detected by MS-based proteomics. **(F)** PRMT5-GFI1 colocalization in non-leukemic thymocytes. Blue (DAPI) = nucleus, green (AF488) = PRMT5, red (AF555) = GFI1. Mean ± SEM (*n* = 3–4); *p* ^*^ ≤ 0.05, *p* ^**^ ≤ 0.01.

**Figure 2:**
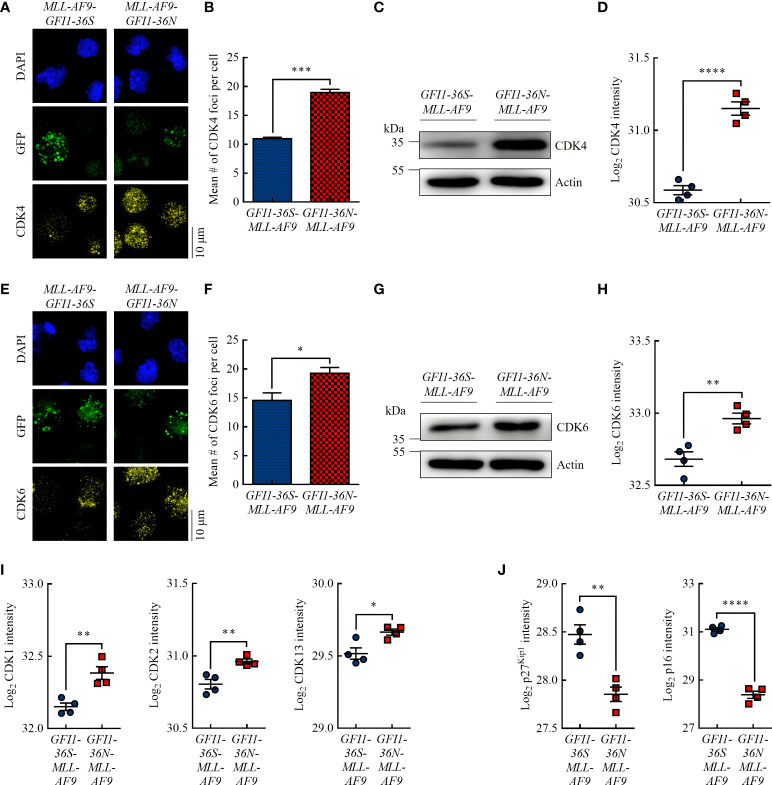
*GFI1-36N* leukemic cells are associated with higher protein levels of the cell cycle kinases CDK4 and CDK6. **(A)** CDK4 foci. Blue (DAPI) = nucleus, green (GFP) = leukemic cells, yellow (AF555) = CDK4. **(B)** Quantification of CDK4 foci. **(C)** CDK4 protein level detected by immunoblotting. **(D)** CDK4 protein intensity detected by MS-based proteomics. **(E)** CDK6 foci. Blue (DAPI) = nucleus, green (GFP) = leukemic cells, yellow (AF555) = CDK6. **(F)** Quantification of CDK6 foci. **(G)** CDK6 protein level detected by immunoblotting. **(H)** CDK6 protein intensity detected by MS-based proteomics. **(I)** CDK1, CDK2, and CDK13 protein intensities detected by MS-based proteomics. **(J)** P27^Kip1^ and p16 protein intensities detected by MS-based proteomics. Mean ± SEM (*n* = 3–4); *p* ^*^ ≤ 0.05, *p* ^**^ ≤ 0.01, *p* ^***^ ≤ 0.001, *p* ^****^ ≤ 0.0001.

To investigate the effect of the *GFI1-36N* SNP on cell cycle, *GFI1-36S* and *GFI1-36N* homozygous lineage-negative progenitor cells were retrovirally transduced with an *MLL-AF9-GFP* construct and transplanted into lethally irradiated mice. When AML symptoms were evident, the mice were euthanized, and the BM cells were isolated and transplanted into sublethally irradiated mice. As soon as these showed the first AML symptoms, the BM cells were removed and used for experiments or further transplantations (**Supplementary Figure 1**). Presence of AML was verified by evaluation of liver and spleen morphology (**Supplementary Figure 2A**), blood count (**Supplementary Figure 2B**), determining the amount of GFP^+^ and c-Kit^+^ cells (**Supplementary Figure 2C**), and by differentiating myeloid cells using CD11b-Gr-1 flow cytometry (**Supplementary Figure 2D**). In addition, GFP was measured by immunofluorescence (**Figures 1C**, **2A**, **E**).

In accordance with previous publications (15, 21), we showed that presence of the *GFI1-36N* SNP was associated with increased proliferation of murine leukemic cells (**Figure 1A**) and with a 2.3-fold higher number of CFUs in CFU assay (**Figure 1B**). A possible explanation for this could be a different activity of the methyltransferase PRMT5. PRMT5 has pro-proliferative and pro-oncogenic functions (22, 23) and, possibly recruited by GFI1, can form a complex with the LIM protein AJUBA and thereby affect chromatin structure (24). Increased PRMT5 protein expression is associated with tumor growth in numerous malignancies by reducing the activity of tumor suppressing miRNAs *via* histone tail modifications (25–27). Using immunofluorescence, we found that *GFI1-36N* leukemic cells had significantly more PRMT5 foci than *GFI1-36S* leukemic cells (**Figures 1C**, **D**). We confirmed that the PRMT5 protein level was increased in presence of the *GFI1-36N* SNP by MS-based proteomics (**Figure 1E**). A greater amount of PRMT5 was localized in the cytoplasmic fraction in leukemic *GFI1-36N* cells than the *GFI1-36S* cells (**Supplementary Figure 3**). GFI1-PRMT5 colocalization could not be shown in leukemic cells because of the numerous PRMT5 and GFI1 foci and the rather planar than dotted PRMT5 staining. Since lower numbers of PRMT5 and GFI1 foci were expected in non-leukemic cells due to fewer DNA damage events and less proliferation, staining was also performed in non-leukemic thymocytes to investigate a possible PRMT5-GFI1 colocalization. We used thymocytes because of their high GFI1 expression (28). As expected, we observed that the number of PRMT5 and GFI1 foci was lower in thymocytes than in leukemic BM cells. Overall, 53.16% ± 6.33% of PRMT5 foci were colocalized with GFI1-36S and 48.81% ± 14.03% with GFI1-36N proteins (**Figure 1F**). No significant differences were observed between *GFI1-36S* and *GFI1-36N* cells.

### *GFI1-36N* leukemic cells are associated with increased CDK levels

Studies have shown that increased PRMT5 protein expression is associated with increased expression of the G_1_ checkpoint proteins CDK4 (29) and CDK6 (30) and is inversely correlated with the CDK inhibitor protein p16 (31). Using immunofluorescence, we demonstrated that *GFI1-36N-MLL-AF9* cells had significantly more CDK4 and CDK6 foci than *GFI1-36S-MLL-AF9* cells (**Figures 2A**, **B**, **E**, **F**). Immunoblotting (**Figures 2C**, **G**) and MS-based proteomics (**Figures 2D**, **H**) showed consistent results. Interestingly, we also demonstrated by MS that in addition to CDK4 and CDK6, the protein levels of other CDKs, namely CDK1, CDK2, and CDK13, were significantly increased in *GFI1-36N* leukemic cells (**Figure 2I**). Correspondingly, the levels of the CDK inhibitor proteins p27^Kip1^ and p16 were significantly decreased in *GFI1-36N-MLL-AF9* cells (**Figure 2J**).

These findings suggest that the *GFI1-36N* genotype is associated with decreased cell cycle arrest control. This might be one reason for the higher proliferation as well as the previously detected genomic instability in *GFI1-36N* leukemic cells.

### *GFI1-36N* leukemic cells are more sensitive to the CDK4/6 inhibitor palbociclib

Given the increased CDK4 and CDK6 protein levels in *GFI1-36N-MLL-AF9* cells, we addressed the question of whether this could be exploited therapeutically. The use of CDK4/6 inhibitors in AML therapy is currently under intense investigation, and initial studies appear promising (32–36). Since no distinction has yet been made with respect to the two *GFI1* variants, we treated *GFI1-36S* and *GFI1-36N* leukemic cells with the selective and clinically established CDK4/6 inhibitor palbociclib.

**Figure 3:**
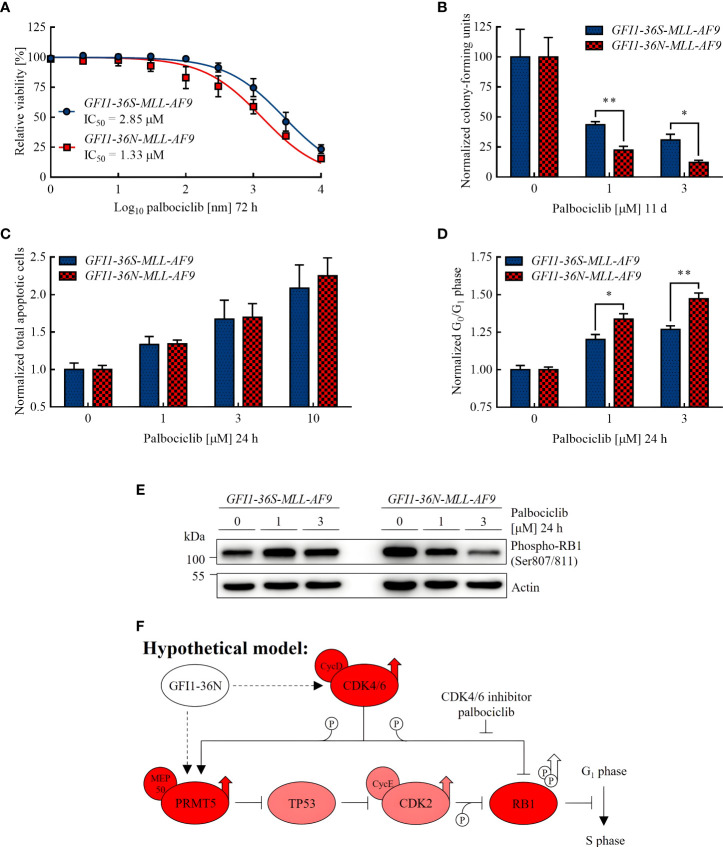
*GFI1-36N* leukemic cells are more sensitive to CDK4/6 inhibition. **(A)** Cell viability after 72 h of palbociclib treatment. **(B)** Normalized CFUs after 11 d of palbociclib treatment. **(C)** Normalized total apoptotic cells after 24 h of palbociclib treatment. **(D)** Normalized cells in G_0_ or G_1_ phase after 24 h of palbociclib treatment. **(E)** Phospho-RB1 protein level detected by immunoblotting after 24 h of palbociclib treatment. **(F)** Hypothetical model of the influence of the GFI1-36N protein on cell cycle regulation: Presence of the *GFI1-36N* variant is correlated with higher CDK4/6 and PRMT5 protein levels leading to RB1 inactivation by phosphorylation and thus cell cycle progression (simplified Figure 1 in Sheppard and AbuHammad 2019 (42) modified according to **Figures 1** and **2** of this manuscript). Mean ± SEM (*n* = 3–6); *p* ^*^ ≤ 0.05, *p* ^**^ ≤ 0.01.

The IC_50_ value of palbociclib approximated in cell viability assay was lower in *GFI1-36N* leukemic cells (1.33 μM) than in *GFI1-36S* leukemic control cells (2.85 μM) (**Figure 3A**). To investigate the differentiation and proliferation abilities of leukemic cells after palbociclib treatment, we performed CFU assays. We found that *GFI1-36N-MLL-AF9* cell populations contained significantly fewer CFUs than *GFI1-36S-MLL-AF9* cell populations after 11 d of palbociclib treatment, compared to the normalized untreated control (**Figure 3B**). However, we did not detect any significant differences in apoptosis rates between *GFI1-36S* and *GFI1-36N* cells, not even when using higher palbociclib concentrations (**Figure 3C**). This suggests that the effect on *GFI1-36N* leukemic cells was not due to an induction of apoptosis. Additionally, we treated *GFI1-36S-MLL-AF9* and *GFI1-36N-MLL-AF9* cells with palbociclib *in vitro* and used these cells for secondary BM transplantation. However, we did not observe *in vivo* AML development (**Supplementary Figure 4**). Evaluating the effect of palbociclib *in vivo* requires a more detailed approach in further studies.

To address the question of whether the higher efficacy of palbociclib was limited to *GFI1-36N* homozygous cells, we also tested *GFI1-36N-MLL-AF9* heterozygous cells still expressing the murine *Gfi1* on one allele, as well as the human chronic myeloid leukemia (CML) cell line K562 transduced with the *GFI1-36N* variant. We found that both *GFI1-36N-MLL-AF9* (**Supplementary Figures 5A**, **B**) and *GFI1-36N*-K562 cells (**Supplementary Figures 6A**–**C**) were more sensitive to CDK4/6 inhibition than *GFI1-36S-MLL-AF9* and *GFI1-36S*-K562 cells. We therefore hypothesize that the therapeutic option described is not restricted to homozygous *GFI1-36N* leukemic cells. For further experiments, we chose to continue using homozygous *GFI1-36S-MLL-AF9* and *GFI1-36N-MLL-AF9* cells.

As palbociclib inhibits transition from G_1_ to S phase and can possibly induce malignant cells to exit the cell cycle (38, 39), we expected more *GFI1-36N* cells in G_0_ or G_1_ phase after treatment if palbociclib had a stronger effect on cells expressing the *GFI1-36N* SNP. By performing cell cycle assays, we confirmed this hypothesis (**Figure 3D**). Furthermore, we demonstrated by immunoblotting that the level of phosphorylated retinoblastoma protein transcriptional corepressor 1 (RB1) was substantially higher in non-treated *GFI1-36N-MLL-AF9* cells (**Figure 3E**), whereas non-phosphorylated RB1 was reduced (**Supplementary Figure 7**). RB1 is a tumor suppressor that is inactivated by phosphorylation by a complex of CDK4 and CDK6 with cyclin D (40). The increased level of phosphorylated and the lower level of non-phosphorylated RB1 in *GFI1-36N* leukemic cells support the hypothesis that cell cycle control mechanisms are disturbed in presence of the *GFI1-36N* variant. This could accelerate cell cycle progression and thus proliferation. Interestingly, we found that the phospho-RB1 level could be reduced by palbociclib treatment primarily in *GFI1-36N* leukemic cells, whereas it remained almost identical in *GFI1-36S* leukemic cells. Correspondingly, a dose-dependent effect was shown only in *GFI1-36N* cells (**Figure 3E**). This supports our observations indicating that *GFI1-36N-MLL-AF9* cells were more sensitive to CDK4/6 inhibition.

Overall, the results presented here show that *GFI1-36N* leukemic cells responded better to palbociclib treatment than *GFI1-36S* leukemic cells, possibly due to the increased proliferation and cell cycle activity as a result of the presence of *GFI1-36N*.

## Discussion

Different malignancies are associated with alterations in CDK activity resulting in cell cycle regulatory disorders (41). CDKs promote cell cycle progression, cell proliferation and thus may be major players in oncogenesis. Therefore, it becomes obvious that inhibition of CDKs is a promising therapeutic approach for treating malignant diseases. In recent years, the first CDK inhibitors were approved for the treatment of advanced breast cancer, but may also provide a therapeutic approach to AML patients (33).

As previously reported by our group, presence of the *GFI1-36N* variant, in contrast to its *GFI1-36S* counterpart, is associated with epigenetic changes leading to decreased repression of oncogenes (10, 21). This promotes leukemogenesis and confers inferior prognosis (12). In line with this, we could show here that the *GFI1-36N* genotype was associated with higher protein levels of the pro-proliferative and pro-oncogenic enzyme PRMT5. Because GFI1 may be responsible for recruitment of the AJUBA-PRMT5 complex to target genes (24), alterations in PRMT5 activity could indicate modified interaction with the respective GFI1 variant. However, this needs to be investigated in more detail in further studies.

Since PRMT5 levels are positively correlated with the levels of CDKs, we explored the presence of other cell cycle changes in *GFI1-36N* leukemic cells and whether these could possibly be exploited as specific targets for novel AML therapies. Indeed, we found that the protein levels of several CDKs were significantly increased in *GFI1-36N-MLL-AF9* leukemic cells, whereas those of the CDKIs p27^Kip1^ and p16 were significantly decreased. Among the largest differences were detected for CDK4 and CDK6, which are the enzymes targeted by clinically approved inhibitors (37). Consequently, we treated *GFI1-36N* and *GFI1-36S* leukemic cells with the established CDK4/6 inhibitor palbociclib. We demonstrated that *GFI1-36N* leukemic cells were more sensitive to palbociclib treatment than *GFI1-36S* leukemic cells and that more *GFI1-36N* cells were in G_0_ or G_1_ arrest after CDK4/6 inhibition. We assume that the observations are not limited to *GFI1-36N* homozygous cells or cells expressing the *MLL-AF9* fusion gene, as heterozygous *GFI1-36N-MLL-AF9* cells as well as *GFI1-36N*-expressing K562 cells were also more susceptible to CDK4/6 inhibition. Certainly, further studies are needed to assess similarities and differences between *GFI1-36N* homozygous and heterozygous cells, especially with regard to proliferation, cell cycle regulation and DNA repair ability.

In addition, we found that the *GFI1-36N* variant was associated with a higher level of phosphorylated and thus inactive RB1. The increased phospho-RB1 level in cells expressing the *GFI1-36N* SNP is related to the increased CDK4/6 and PRMT5 protein levels detected in this work, as these enzymes are responsible for RB1 phosphorylation (**Figure 3F**) (42). In *GFI1-36N-MLL-AF9* cells, RB1 phosphorylation seems to be reversible by CDK4/6 inhibition. This would decrease proliferation of the *GFI1-36N* leukemic cells and may provide an explanation why these cells were more responsive to palbociclib treatment than *GFI1-36S* leukemic controls.

As we did not detect any significant differences in apoptosis rates between *GFI1-36S* and *GFI1-36N* cells after treatment, we assume that the higher efficacy on *GFI1-36N* leukemic cells was not due to increased apoptosis, but due to its effect on cell proliferation. One possible explanation may be that CDK4/6 inhibitors primarily force *GFI1-36N* leukemic cells to exit the cell cycle since the mechanism of initiating senescence or quiescence after CDK4/6 inhibition has been previously described by numerous groups and is currently an active field of research (39, 43–45).

Further investigation is needed to better understand how presence of *GFI1-36N* and its transcriptional product leads to deregulation of CDK4 and CDK6, especially since we did not find any available CHIP-Seq databases showing that GFI1 binds to the promoter of PRMT5, CDK4, or CDK6. This suggests that the increased CDK4 and CDK6 levels in *GFI1-36N* leukemic cells may be not due to direct interaction with GFI but due to indirect effects, possibly mediated by other proteins not addressed in this manuscript. Investigation of the underlying mechanism could be considered in additional studies. Moreover, it would be interesting to explore in more detail whether the therapeutic approach described in this manuscript is transferable to other hematologic neoplasms expressing the *GFI1-36N* variant (46).

In summary, we demonstrated that murine *GFI1-36N-MLL-AF9* cells were associated with increased PRMT5, CDK4, and CDK6 protein levels leading to phosphorylation and thus inactivation of RB1. One possible consequence is a disturbed cell cycle control and therefore an increased proliferation of *GFI1-36N* leukemic cells. This could explain why *GFI1-36N* leukemic cells were more sensitive to palbociclib treatment compared to *GFI1-36S* leukemic control cells. *GFI1-36N* would potentially be a promising marker for a specific subset of AML patients who might particularly benefit from therapy with CDK4/6 inhibitors.

## Data availability statement

The raw data supporting the conclusions of this article will be made available by the authors, without undue reservation.

## Ethics statement

The animal study was reviewed and approved by the local ethics committee of North Rhine-Westphalia.

## Author contributions

JV and KS performed research, designed the study, analyzed data, and wrote the manuscript. DF contributed to experiments, designed the study, and analyzed data. FN and JH took immunofluorescence pictures and contributed to experiments. PB, LL, XX, and PP contributed to experiments. HA and BO did critical revision of the manuscript. GL supported research and did critical revision of the manuscript. AJ contributed to experiments and analyzed data. CK designed the study, analyzed data, wrote the manuscript, and provided funding. The final version of the manuscript has been read and approved by all named authors.

## Funding

The work was funded by the German José Carreras Foundation (DJCLS, 17R/2018), and partially by the German Cancer Aid (DKH, 70112392), the German Research Foundation (DFG, KH331/2-3), and the Interdisciplinary Center for Clinical Research Münster (IZKF, Kha2/002/20). JV was supported by fellowships of the Jürgen Manchot Foundation and the Medical College Münster (MedK). AJ was supported by the Max Planck Society for the Advancement of Science and the German Cancer Research Center (DKFZ).

## Acknowledgments

The authors thank the employees of the Central Animal Facility (ZTE) of the Faculty of Medicine at the University of Münster for taking care of the mice used in the experiments. We thank Hannelore Leuschke and Dagmar Clemens for genotyping mice and their technical assistance, as well as Dr. Kay Jüngling, Institute of Physiology I, University of Münster, for helping with immunofluorescence analysis.

## Conflict of interest

FN was employed by evorion biotechnologies GmbH.

The remaining authors declare that the research was conducted in the absence of any commercial or financial relationships that could be construed as a potential conflict of interest.

## Publisher’s note

All claims expressed in this article are solely those of the authors and do not necessarily represent those of their affiliated organizations, or those of the publisher, the editors and the reviewers. Any product that may be evaluated in this article, or claim that may be made by its manufacturer, is not guaranteed or endorsed by the publisher.

## Abbreviations

AML, acute myeloid leukemia; BM, bone marrow; CDK, cyclin-dependent kinase; CDKI, cyclin-dependent kinase inhibitor protein; CFU, colony-forming unit; CML, chronic myeloid leukemia; GFI1, growth factor independence 1; MS, mass spectrometry; N, asparagine; PRMT5, protein arginine N-methyltransferase 5; RB1, retinoblastoma protein transcriptional corepressor 1; S, serine; SNP, single nucleotide polymorphism
