# Supplementary material for: Presence of the GFI1-36N single nucleotide polymorphism enhances the response of MLL-AF9 leukemic cells to CDK4/6 inhibition
Source: Front Oncol. 2022 Aug 8;12:903691. doi: 10.3389/fonc.2022.903691 (PMC9393725; doi:10.3389/fonc.2022.903691)
Supplement: Supplementary file 1 [file DataSheet_1.pdf]

Supplementary figures

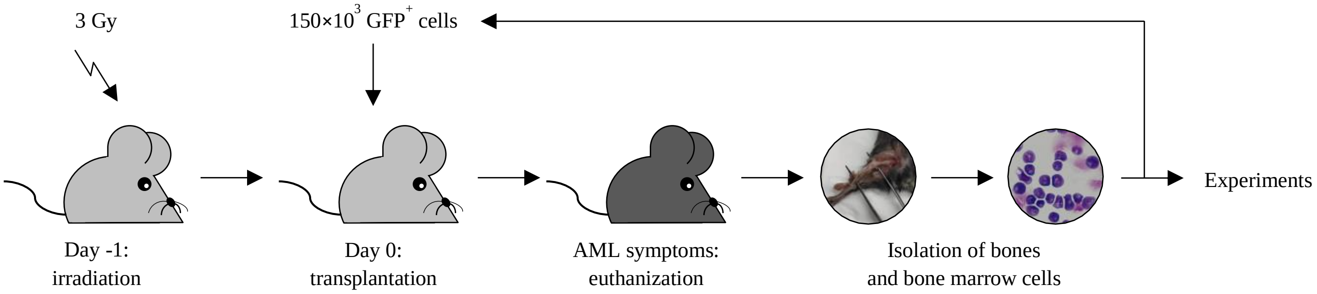

SUPPLEMENTARY FIGURE 1 | Secondary BM transplantation. Mice were irradiated before transplanting leukemic BM cells. When AML symptoms were evident, the mice were sacrificed, the bones were removed, and the BM cells were isolated.

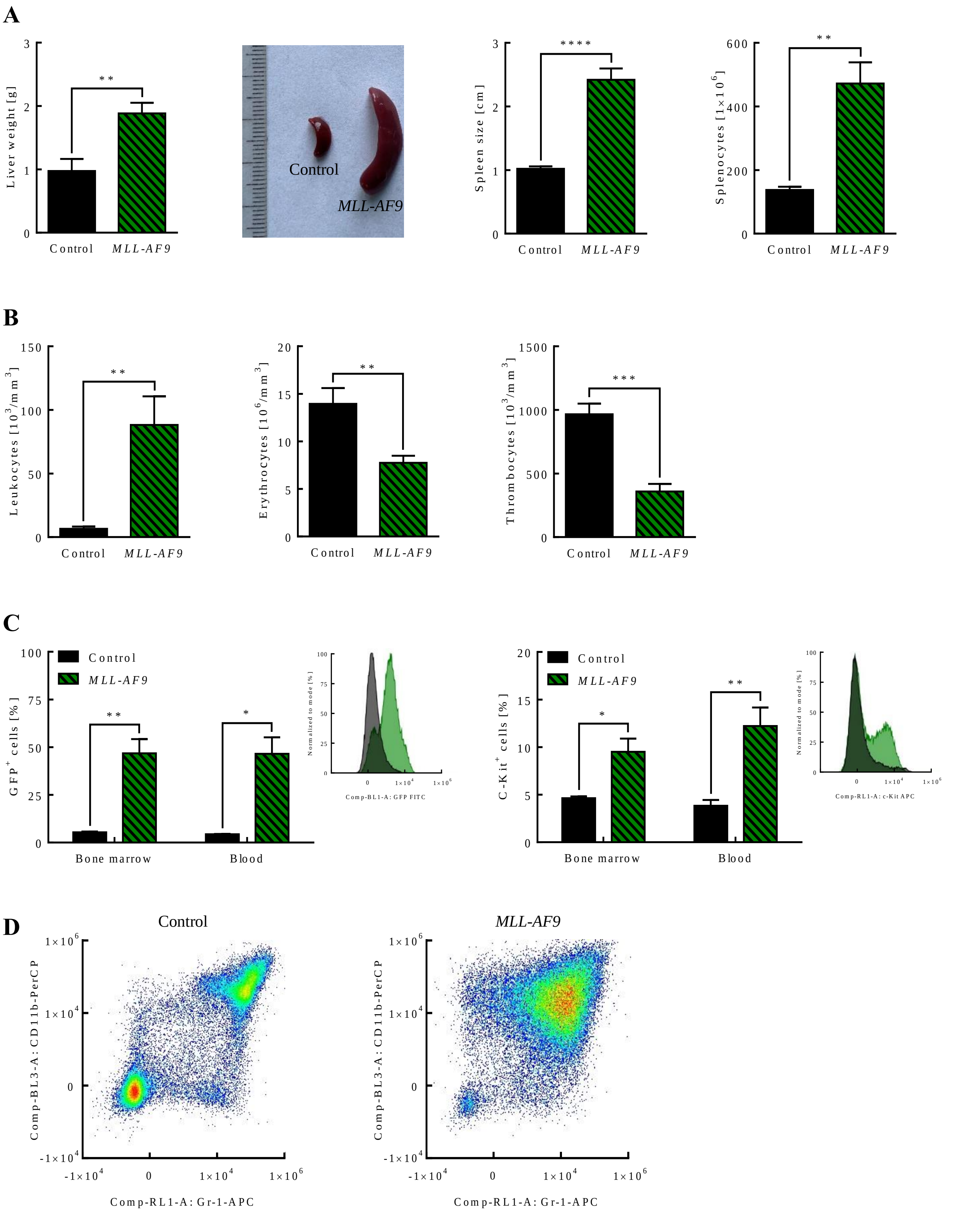

SUPPLEMENTARY FIGURE 2 | Distinction between *MLL-AF9* leukemic cells and non-leukemic control cells. **(A)** Hepatosplenomegaly in leukemic mice. **(B)** Leukocytosis, erythrocytopenia, and thrombocytopenia in leukemic mice. **(C)** GFP$^+$ and c-Kit$^+$ cells in flow cytometry. **(D)** Gr-1-CD11b co-staining in non-leukemic and leukemic BM cells in flow cytometry. Mean ± SEM (n = 5–10); $p^* \leq 0.05$, $p^{**} \leq 0.01$, $p^{***} \leq 0.001$, $p^{****} \leq 0.0001$.

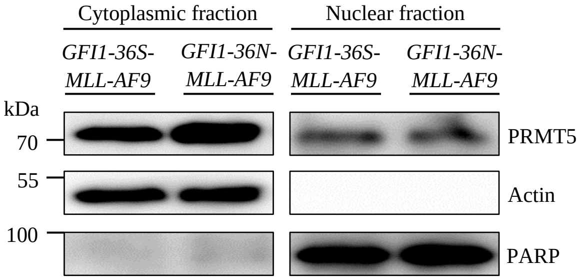

SUPPLEMENTARY FIGURE 3 | Cytoplasmic and nuclear PRMT5 protein levels detected by immunoblotting. $n = 3$.

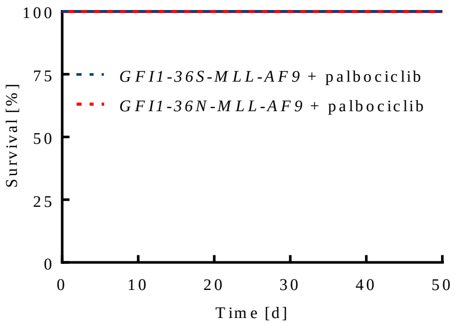

SUPPLEMENTARY FIGURE 4 | Kaplan-Meier survival curves of mice transplanted with cells incubated for 48 h in 0.3 µM palbociclib. $n = 6$.

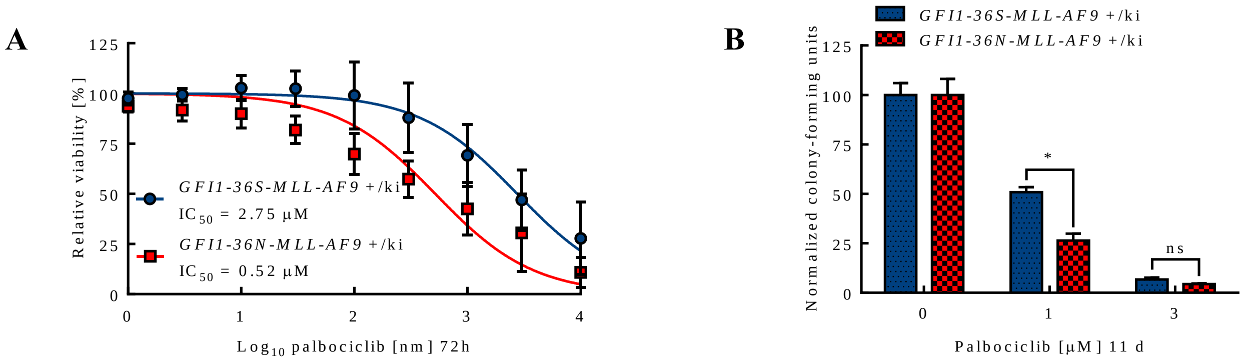

SUPPLEMENTARY FIGURE 5 | Heterozygous *GFI1-36N-MLL-AF9* cells are more sensitive to CDK4/6 inhibition. **(A)** Cell viability after 72 h of palbociclib treatment. **(B)** Normalized CFUs after 11 d of palbociclib treatment. Mean ± SEM (n = 2–4); ns = not significant, $p^{*} \leq 0.05$.

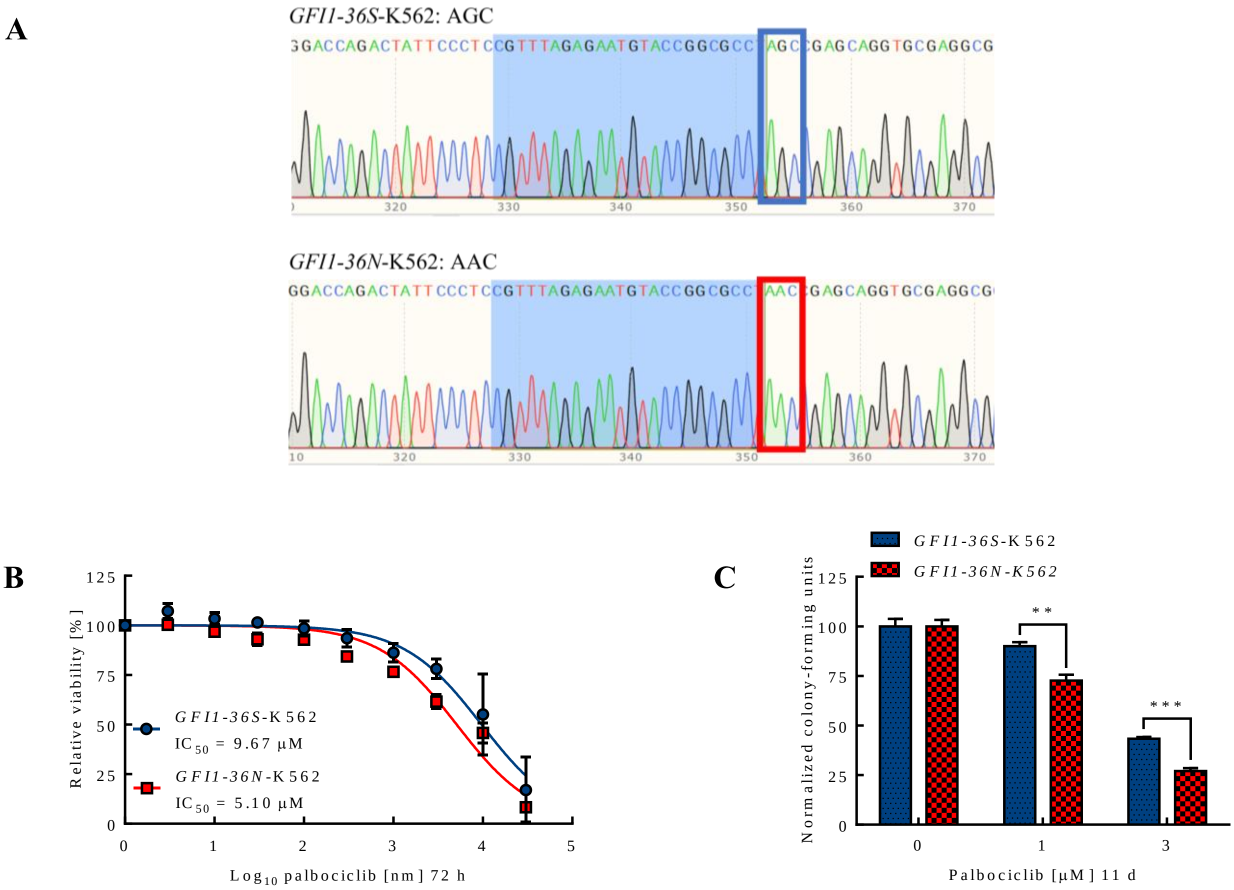

SUPPLEMENTARY FIGURE 6 | *GFI1-36N*-K562 cells are more sensitive to CDK4/6 inhibition. **(A)** Single cell sequencing of *GFI1-36S*-K562 and *GFI1-36N*-K562 cells. **(B)** Cell viability after 72 h of palbociclib treatment. **(C)** Normalized CFUs after 11 d of palbociclib treatment. Mean ± SEM (n = 2–3); $p^{**} \leq 0.01$, $p^{***} \leq 0.001$.

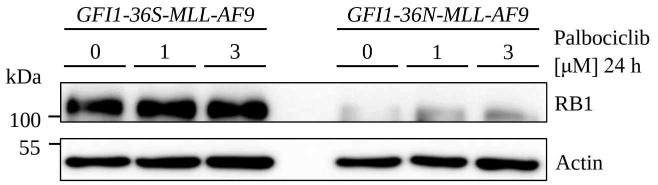

SUPPLEMENTARY FIGURE 7 | RB1 protein level detected by immunoblotting after 24 h of palbociclib treatment. n = 3.

Supplementary methods

1 Distinction between *MLL-AF9* leukemic and non-leukemic cells

1.1 Liver and spleen morphology

The liver was removed and weighed. The spleen was removed, and the size was measured. It was then homogenized and resuspended in 10 ml FACS buffer (2% FCS + 1% P/S in PBS) to count the number of cells in a Neubauer hemocytometer.

1.2 Blood count

Approximately 200 µl of blood was collected from the right ventricle of the mouse heart and transferred to an EDTA-coated tube (Greiner Bio-One, Kremsmünster, Austria). The blood was analyzed in a scil vet abc hematology analyzer (Scil, Viernheim, Germany).

1.3 GFP measurement and antigen staining

In addition to the percentage of GFP^+ cells, the expression of seven antigens was determined by flow cytometry. Six of the seven antigens were co-stained: Gr-1 as a marker for granulocytes and CD11b as a marker for macrophages, B220 as a marker for B cells and Ter119 as a marker for erythroid differentiation stages as well as CD4 as a marker for T helper cells and CD8 as a marker for cytotoxic T cells. Single staining was performed for c-Kit as a marker for hematopoietic and leukemic stem cells. An unstained sample was used to measure the GFP signal.

Gr-1-APC/CD11b-PerCP (108411/101229, BioLegend, San Diego, CA, United States), Ter119-APC/B220-PerCP (116211/103235, BioLegend), and CD8a-APC/CD4-PerCP (100711/100537, BioLegend) stainings were performed in bone marrow (BM) cells. Ter119-APC/B220-PerCP and CD8a-APC/CD4-PerCP stainings were used to exclude presence of acute lymphoblastic leukemia (ALL) or mixed phenotype acute leukemia (MPAL) and are therefore not shown in the manuscript. C-Kit-APC (105811, BioLegend) staining and GFP detection were performed in BM and blood cells.

10 µl BD Fc block (Becton Dickinson, Franklin Lakes, NJ, United States) was added to 0.25×10^6 cells. Samples were incubated on ice for 4 min and then washed once in FACS buffer. This was followed by single or double staining with the antibodies listed above. They were diluted in FACS buffer to a concentration of 0.2 µg/ml. 80 µl of the antibodies were added to each sample and incubated on ice for 10 min. The cells were then rewashed in FACS buffer and analyzed in an Attune NxT acoustic focusing cytometer using Attune NxT version 3 software (Thermo Fisher Scientific, Waltham, MA, United States).

2 Immunoblotting of nuclear and cytoplasmic fractions

Nuclear and cytoplasmic protein fractions were separated using NE-PER nuclear and cytoplasmic extraction reagents (Thermo Fisher Scientific) according to the manufacturer's instructions. Immunoblotting was performed as described in the methods. PARP was chosen as the nuclear reference protein. The PARP antibody (9642, Cell Signaling Technology, Danvers, MA, United States) was diluted to a concentration of 1:1,000 in 5% milk in TBS-T.

3 Generation of *GFI-36N*-K562 cells

Human K562 cells were obtained from DSMZ (Braunschweig, Germany). They were maintained in

RPMI with 10% FBS and 1% P/S at 37 °C and 5% CO_2. To generate *GFI1-36N* cells, two lentiviral plasmids were used. Lenti-hyeA3A-BE4max was modified from Lenti-117G-hyeA3A-BE4max, which was a gift from Dali Li (1) (plasmid #157946, Addgene, Watertown, MA, United States), and tet-pLKO-sgRNA-puro was a gift from Nathanael Gray (2) (plasmid #10432, Addgene). Lentiviral production and transduction were performed as described (3). The infected K562 cells were selected by GFP sorting and puromycin selection. Single clones were isolated by limiting dilution in 96-well cell culture plates. The cells were expanded for 14 d before genomic DNA sequencing (Supplementary Figure 6A).

Supplementary references

1. Zhang X, Chen L, Zhu B, Wang L, Chen C, Hong M, et al. Increasing the efficiency and targeting range of cytidine base editors through fusion of a single-stranded DNA-binding protein domain. Nat Cell Biol. 2020;22(6):740–50.

2. Huang HT, Seo HS, Zhang T, Wang Y, Jiang B, Li Q, et al. MELK is not necessary for the proliferation of basal-like breast cancer cells. eLife. 2017;6.

3. Marneth AE, Botezatu L, Hönes JM, Israël JCL, Schütte J, Vassen L, et al. GFI1 is required for RUNX1/ETO positive acute myeloid leukemia. Haematologica. 2018;haematol.2017.180844.
